# Multi-omics analysis identifies PPARα as a key inhibitor of hepatocyte ferroptosis in sepsis-associated liver injury

**DOI:** 10.1371/journal.pone.0338591

**Published:** 2026-02-19

**Authors:** Shi-Ling He, Jian-Chuan Lin, Gao-Fang Wu, He-fan He

**Affiliations:** 1 Department of Anesthesiology, The Second Affiliated Hospital of Fujian Medical University, Quanzhou, China; 2 Department of Anesthesiology, The First Hospital of Quanzhou Affiliated to Fujian Medical University, Quanzhou, China; 3 Department of Anesthesiology, Shishi General Hospital, Quanzhou, China; Helwan University, EGYPT

## Abstract

**Background:**

Sepsis-associated liver injury (SLI) increases the risk of death in septic patients, and a primary pathological alteration in sepsis is ferroptosis in the liver. However, the specific mechanism of its occurrence remains largely unclear. Our work is to validate key pathways connected with ferroptosis in SLI and elucidate potential pharmaceutical targets involved in this process.

**Methods:**

To confirm targets related to SLI, we screened three SLI microarray datasets (GSE23767, GSE40180 and GSE104342) from the GEO database and obtained the ferroptosis-related differentially expressed genes (FRDEGs). A functional enrichment analysis of FRDEGs was performed. The protein–protein interaction network was used to visualize the interactive relationship of FRDEGs. Additionally, the potential biological functions and enrichment pathways of FRDEGs were elucidated through GO and KEGG analysis. Furthermore, we obtained the single-cell dataset (GSE238000) from liver tissue from GEO database to determine the series of cell subtype mainly expressing target molecules. Finally, we performed a serial of in vivo and in vitro experiments to further validate the findings of bioinformatic analysis.

**Results:**

A total of 51 genes that are expressed differently in SLI involve ferroptosis. These genes are involved in negative regulation of apoptotic process, the endoplasmic reticulum, and identical protein binding. The KEGG pathway study revealed that they were mainly involved in the PPAR signaling pathway. Among three isoforms of PPAR family, PPARα is most abundant in the liver. We then observed that it was significantly downregulated in hepatic tissue of SLI mice and PPARα agonist WY-14643 effectively blocked sepsis-induced hepatic ferroptosis. Subsequently, single-cell analysis demonstrated that PPARα is predominantly expressed in hepatocytes, which was downregulated in LPS-treated THLE-2 cells. Notably, consistent with the in vivo results, PPARα activator WY-14643 also significantly alleviated LPS-induced ferroptosis in THLE-2 cells.

**Conclusion:**

The validation of ferroptosis-related pathway PPARα in this study greatly deepens our understanding of the potential mechanism of SLI, and provide promising therapeutic targets for the disease.

## Introduction

Sepsis refers to a deadly organ dysfunction resulting from the dysregulation of host response to infection. It is one of the major causes resulting in mortality in critical patients in intensive care unit (ICU). Liver is one of the most vulnerable organs and can be impaired at any stage during sepsis [1]. This impairment causes liver dysfunction and even failure, directly leading to sepsis progression and death [2]. Hence, a dysfunctional liver is probably a core factor for the initiation and development of multi-organ functional disturbance. It’s reported that the death rate associated with sepsis-associated liver injury (SLI) occurs in around 54% to 68% of septic patients, obviously surpassing the death rate of septic patients with pulmonary injury, which is among the most frequently impacted organs in sepsis [2]. The primary manifestations of SLI include cholestasis, coagulopathy, hypoxic hepatitis, and damage of metabolism-related protein synthesis [3]. Regrettably, the pathophysiology of SLI remains to be fully understood. Thus, there are currently no effective therapeutic options to cure this disease.

Recent research on the pathogenesis of SLI primarily emphasizes the role of microcirculatory and immune system disorders that contribute to mitochondrial dysfunction and cell death [4,5]. Despite this progress, the precise mechanisms underlying the onset and progression of SLI remain poorly understood. In recent years, an increasing number of studies have investigated the impact of microelement metabolic disorders on the development of SLI [6]. Ferroptosis is a newly-found type of programmed cell death, characterized by unique genetic, morphological, biochemical features. Intracellular dysregulated iron metabolism and lipid peroxidation are two indispensable processes of ferroptosis, which can contribute to membrane disruption and cellular contents outflow, finally resulting in cell death [7]. Emerging evidence has indicated that ferroptosis is involved in a variety of pathophysiological changes and related to a variety of diseases, including liver disease [8], lung adenocarcinoma [9], diabetes complications [10], and sepsis-induced cardiac injury [11], etc. Ferroptosis can exacerbate the inflammatory response, ultimately leading to increased organ damage. Thus, besides the aforementioned diseases, ferroptosis also plays a crucial role in SLI [12]. However, the specific mechanisms and genes related to the initiation of ferroptosis in SLI have not been revealed.

Prostaglandin-endoperoxide synthase (PTGS), commonly referred to as cyclooxygenase, plays a crucial role in prostaglandin synthesis, exhibiting both dioxygenase and peroxidase activities [13]. PTGS2 is primarily responsible for generating proinflammatory prostaglandins that can lead to oxidative stress and lipid peroxidation, both of which are key features of ferroptosis. Selective PTGS2 inhibitors, including nonsteroidal anti-inflammatory drugs (NSAIDs) such as celecoxib, have been developed for clinical use in managing inflammation and pain [14]. Thus, PTGS2 has been identified as a marker of ferroptosis.

In this study, we employed bioinformatics technology to quickly excavate ferroptosis-related differentially expressed genes (FRDEGs). Then, we performed functional and pathway enrichment analysis to explore the potential functions and pathways of FRDEGs. Afterward, single-cell analysis technique was used to determine the cellular distribution of target genes in liver tissue. Ultimately, we validated the underlying mechanism of sepsis-induced hepatic ferroptosis via series of in vivo and in vitro experiments.

## Methods

**Datasets and processing** We downloaded SLI-related microarray data from the Gene Expression Omnibus database (GEO) for bioinformatics analysis. In this study, we included three microarray datasets for subsequent analyses, including GSE23767, GSE40180 and GSE104342. A meticulous pre-processing procedure was performed to ensure the usability and integrity of the raw data. To stabilize the difference across different expression levels, we transformed the microarray data to logarithmic values using log2 transformation method. After this transformation, we converted probe IDs to corresponding gene symbols. If multiple probe IDs represented a gene, we calculated their average as the final gene expression value. Subsequently, we employed the Combat algorithm in the “sva” R package to remove batch effects, then creating a combined dataset.

### Identification of differential expression genes (DEGs) and ferroptosis-related differentially expressed genes (FRDEGs)

DEGs were validated using the “limma” R package, filtering for |Log2FC| > 0.5 and P value<0.05. Visualization was conducted using “ggplot” R package for a volcano plot and “heatmap” R package for a heatmap. Afterward, we collected and collated the ferroptosis-related genes (FRGs) from the “FerrDb” database and Genecards database. A Venn plot depicting the intersection of DEGs and FRGs was generated through the online tool Venny 2.1 (https://bioinfogp.cnb.csic.es/). Additionally, we uploaded FRDEGs to STRING database. Thereafter, Cytoscape software was employed to construct a protein-protein interaction (PPI) network, which permitted us to reveal the interactions of FRDEGs.

**Functional enrichment analysis and protein-protein interaction network** To elucidate the functions and pathways of FRDEGs, we used the DAVID website (http://david.ncifcrf.gov/home.jsp) to perform enrichment analysis, including GO terms and KEGG pathway enrichment analyses. GO analysis consisted of Biological Processes (BP), Cell Components (CC), and Molecular Functions (MF) terms. We set P < 0.05 as the statistical significance threshold for all GO enrichment and KEGG pathway analyses.

**Single-cell RNA sequencing (scRNA-seq) analysis**We then obtained the single-cell dataset (GSE238000) from liver tissue from GEO database. The expression profiles of key genes were punctiliously analyzed via scRNA-seq analysis. To ensure data integrity and quality, we performed the initial quality control procedures to filter the cells using several rigorous criteria. Cells with detected genes below 200 or exceeding 10,000, total counts fewer than 100 or surpassing 30,000, and the proportion of mitochondrial genes exceeding 20% were removed from further analysis. Subsequently, the data underwent normalization employing the NormalizeData function and ScaleData function. An elbow plot was generated to validate the appropriate principal components (PCs) and Uniform Manifold Approximation and Projection (UMAP) method was used to conduct clustering analysis on dimensionality-reduced cells, with the resolution set at 0.1. Additionally, we annotated these obtained cell clusters using SingleR method. Meanwhile, we obtained the marker genes of each cell subtype from literatures to validate the annotation results of SingleR method [15,16].

**Establishment of SLI mice model** Male C57BL/6 mice (20–25g) aged 6–8 weeks were raised in appropriate humidity, temperature and illumination cycle, with free access to food and water. The SLI model was established through intraperitoneally injecting lipopolysaccharide (LPS, 25 mg/kg, Sigma-Aldrich, USA) [17–19]. Control mice were administered intraperitoneal saline. All mice were euthanized 24 h by intraperitoneal injection of 1% pentobarbital sodium to harvest the liver tissues for subsequent experiments. All the animal procedures performed in this work performed conformed to the requirements set forth by the Ethics Committee of Second Affiliated Hospital of Fujian Medical University (2025206). Meanwhile, we have completed the PLOSOne_Human_Subjects_Research_Checklist (S1 Table).

**Cell culture and processing** THLE-2 cells (Human liver epithelial, Anweisic, Shanghai, China) were cultured in BEBM (Lonza, Basel, Switzerland) supplemented with 10% fetal bovine serum (FBS) and 2% penicillin/streptomycin. Then, cells were incubated at 37°C in a 5% CO_2_ environment. For establishing an in vitro model, the cells were treated with 1 μg/mL LPS to create an activated inflammatory cell model. 24 hours after stimulation, THLE-2 cells were collected to biochemical analysis.

#### Quantitative reverse transcription-Polymerase Chain Reaction (qRT-PCR).

Total RNA from liver tissues and THLE-2 hepatocytes were extracted using total RNA extraction reagent (Biosharp, China). cDNA was then synthesized utilizing the cDNA Synthesis Kit (Takara, Japan). Quantitative real-time PCR was executed employing SYBR Green PCR SuperMix (Servicebio, China). The relative expression value of target genes unified to GAPDH was calculated. All primer sequences utilized in this work were listed in Table 1. Data were analyzed according to the sample threshold cycle (Ct) value from three independent experiments.

**Table 1:** mRNA-specific primers of genes.

Gene	Primer	Sequence (5’-3’)
GAPDH	F	GGCAAATTCAACGGCACAGTCAAG
	R	TCGCTCCTGGAAGATGGTGATGG
IL-1B	F	CACTACAGGCTCCGAGATGAACAAC
	R	TGTCGTTGCTTGGTTCTCCTTGTAC
IL-6	F	CTTCTTGGGACTGATGCTGGTGAC
	R	TCTGTTGGGAGTGGTATCCTCTGTG
TNF	F	CGCTCTTCTGTCTACTGAACTTCGG
	R	GTGGTTTGTGAGTGTGAGGGTCTG
PTGS2	F	CTGGTGCCTGGTCTGATGATGTATG
	R	GGATGCTCCTGCTTGAGTATGTCG
PPARα	F	TGCAAACTTGGACTTGAACGACC
	R	CCATGATGTCACAGAACGGCTT
PPARβ/δ	F	GAACCGCAACAAGTGTCAGTA
	R	GTAGGCGTTGTAGATGTGCTTAG
PPARγ	F	AGCCCTTTGGTGACTTTATGG
	R	CAGCAGGTTGTCTTGGATGT

**Western blotting analysis** Total proteins were extracted using RIPA lysate (Beyotime, P0013B) containing protease inhibitor, phenylmethylsulfonyl fluoride (PMSF), and phosphatase inhibitor (100:1:1:1) on ice. After determining the protein concentration using bicinchoninic acid (BCA) method, the extracted proteins were subjected to 10% sodium dodecyl sulfate-polyacrylamide (SDS) gel electrophoresis and transferred to a Polyvinylidene Fluoride (PVDF) membrane. To examine the immunoreactive band signal, the membranes were incubated with the primary antibody against PTGS2 (1:1000, Affinity), TFRC (1:1000, ABclonal), GPX4 (1:1000, ABclonal), PPARα (1:1000, ABclonal), and GAPDH (1:10000, Affinity) at 4°C overnight and then incubated with the secondary antibody at room temperature for 1 h. Lastly, the immunoreactive bands were visualized through the enhanced chemiluminescence (ECL) (Biosharp, BL520A) system and the BioRad imaging system. The protein bands were analyzed by the Image J software normalized to GAPDH as a reference. The Original WB blots can be found in (S1 Fig) file.

**Measurement of GSH and MDA** Supernatant was collected after centrifugation [29]. The GSH and MDA detection kits (Solarbio, Beijing, China) were employed to detect the content of GSH and MDA according to manufacturer’s instructions. Data were analyzed from three independent experiments.

**Iron Assay** The iron concentration in THLE-2 cells was examined using FerroOrange (Dojindo, Japan) staining. The cells were grown in conditioned medium in 35 mm dishes and cultured overnight at 37°C in a 5% CO_2_ incubator. Afterward, the cells were rinsed and incubated with 1 µmol/L FerroOrange working solution for 30 minutes. Finally, Fe^2+^ fluorescence intensity of the cells was observed under an inverted confocal microscopy (Nikon).

**Statistical analysis** All statistical analyses were performed using GraphPad Prism 8.0 (GraphPad Inc., San Diego, USA) and R software. Data are presented as mean ± SD. The Shapiro-Wilk test was used to assess data normality. For normally distributed data, Student’s t-test (two-tailed) was applied for comparisons between two groups when assumptions were met. For comparisons among three or more groups, one-way ANOVA was conducted, followed by Tukey’s post-hoc test for multiple comparisons to identify specific group differences. Multiple comparisons were adjusted per experiment using the post-hoc test to control for Type I error. For non-normally distributed data, the Mann-Whitney test (for two groups) or Kruskal-Wallis H test (for three groups) was used. The Kaplan-Meier method was used to analyze mouse survival rates. A p-value of < 0.05 was considered statistically significant.

## Results

**Fig 1:**
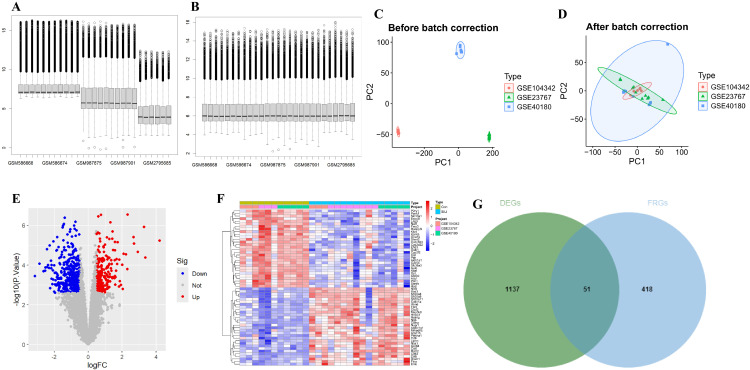
Identification of differentially expressed genes (DEGs). (A) Gene expression level statistics of the integrated dataset before removed batch effect. (B) Gene expression level statistics of the integrated dataset after removed batch effect. (C) principal component analysis (PCA) plot of the integrated dataset before removed batch effect. (D) PCA plot of the integrated dataset after removed batch effect. (E) The volcano plot of DEGs expression. (F) Heatmap of top 30 DEGs expression. (G) Venn plot showing the intersected genes between DEGs and ferroptosis-related genes. FC, fold change.

**Screening of DEGs in SLI** Three microarray datasets (GSE23767, GSE40180 and GSE104342) from the GEO database were used, and 11 control and 16 SLI samples were obtained. To ensure precise data processing, batch effects among three datasets were removed (Fig 1A–D). Boxplot plots (Fig 1A, 1B) and PCA plots (Fig 1C, 1D) demonstrated the successful removal of the batch effects. Afterward, through strict statistical criteria (P < 0.05 and |log2FC| > 0.5), 1188 DEGs were confirmed (S2 Table), including 479 up-regulated genes and 709 down-regulated genes, presented in the volcano plot (Fig 1E). The top 25 up-regulated and 25 down-regulated genes were then visualized via a heatmap (Fig 1F).

**Fig 2:**
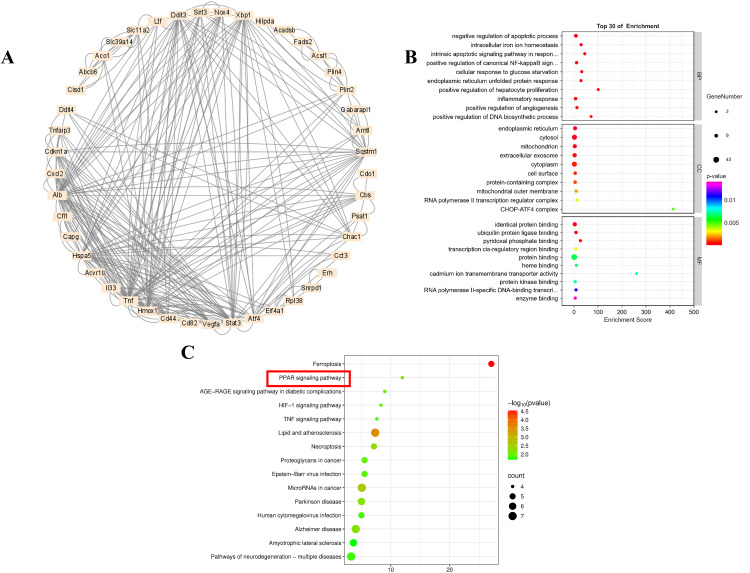
Functional annotation of ferroptosis-related differentially expressed genes (FRDEGs). (A) Protein-protein interaction (PPI) network of FRDEGs. (B) Gene ontology (GO) enrichment analysis; (C) Kyoto Encyclopedia of Genes and Genomes (KEGG) enrichment analysis.

**Screening of FRDEGs in SLI** After acquiring 469 FRGs from the “FerrDb” database and GeneCards database, we intersected them with 1188 DEGs to obtain 51 FRDEGs (Fig 1G) (S3 Table). Among them, 19 were downregulated and 29 were upregulated. Subsequently, we imported these FRDEGs into STRING website to construct a PPI network and used Cytoscape software to visualize their interactions (Fig 2A).

### Functional annotation analysis

The potential biological functions and enrichment pathways of 51 FRDEGs were elucidated through GO and KEGG analysis. GO analysis consisted of three categories: biological process (BP), cellular component (CC), and molecular function (MF). We found that the FRDEGs were significantly enriched in several BP terms involved in negative regulation of apoptotic process, intracellular iron ion homeostasis, intrinsic apoptotic signaling pathway in response to endoplasmic reticulum stress, positive regulation of canonical NF-kappaB signal transduction, and cellular response to glucose starvation; those under CC were significantly associated with the endoplasmic reticulum, cytosol, mitochondrion, extracellular exosome, and cytoplasm, whereas those under MF were significantly correlated with identical protein binding, ubiquitin protein ligase binding, pyridoxal phosphate binding, transcription cis-regulatory region binding, and protein binding (P < 0.05) (Fig 2B) (S4 Table). The top 10 KEGG terms are shown in Fig 2C, including ferroptosis, atherosclerosis (lipid), cancer (microRNAs and proteoglycans), neurodegenerative disorders (Parkinson’s and Alzheimer’s diseases), diabetes (AGE-RAGE signalling) PPAR signaling, necroptosis, and Epstein-Barr virus infection (S5 Table). These findings indicated that PPAR signaling pathway exhibited correlation with SLI.

### Validation of key gene involved of PPAR signaling pathway in SLI

**Fig 3:**
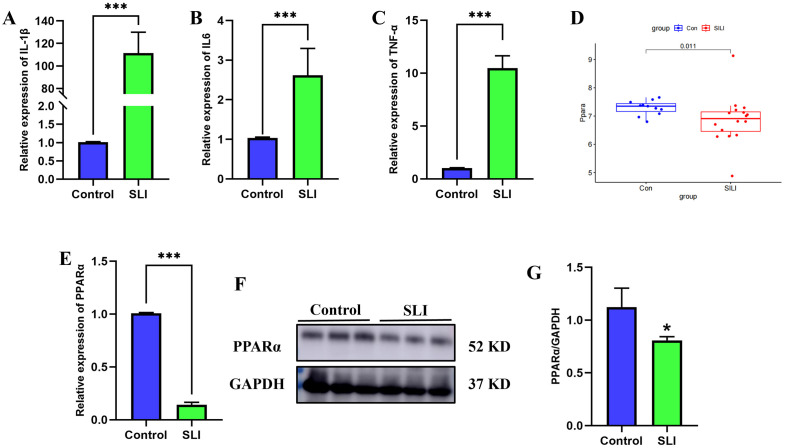
The expression of PPAR α was validated by RT-qPCR and WB in SLI mice. (A-C) The mRNA levels of proinflammatory cytokines (n = 3). (D) Expression validation of PPARα in the integrated dataset. **(E)** The mRNA level of PPARα (n = 3). (F-G) The protein level of PPARα (n = 3). ***, p < 0.001, compared with control group. Data are shown as mean ± standard deviation (SD), and n represents the number of mice in each group.

To confirm the results from KEGG analysis, we first evaluated the expression of key genes of PPAR signaling pathway using our merged dataset. There are three isoforms of PPAR family, including PPARα, PPARβ, and PPARγ. The immunohistochemical results from Human Protein Atlas database (https://www.proteinatlas.org/) indicated that the expression level of PPARα is most abundant in the liver, when compared with other two isoforms (S2 Fig). We then validated the expression difference of three isoforms in liver tissues of SLI mice and control mice. We constructed the SLI mice model and found that the levels of proinflammatory cytokines (IL-1β, IL6, and TNFα) mRNA in SLI mice were significantly higher than in control mice (P < 0.05) (Fig 3A–C). We then validated the expression difference of three isoforms using GEO database and qPCR. The results indicated that compared to the other two isoforms, only the expression of PPARα shows a consistent trend under multiple validations, validating the rationale for selecting PPARα as the experimental target (P < 0.05) (Figs 3D, E, S3). Additionally, we further examined the protein expression profiles of PPARα in liver tissue of mice treated by LPS. PPARα was lowly expressed in the LPS-treated liver tissue versus PBS-treated liver tissue (P < 0.05) (Fig 3F, G).

### PPARα significantly inhibited the LPS-induced hepatic ferroptosis in SLI mice

**Fig 4:**
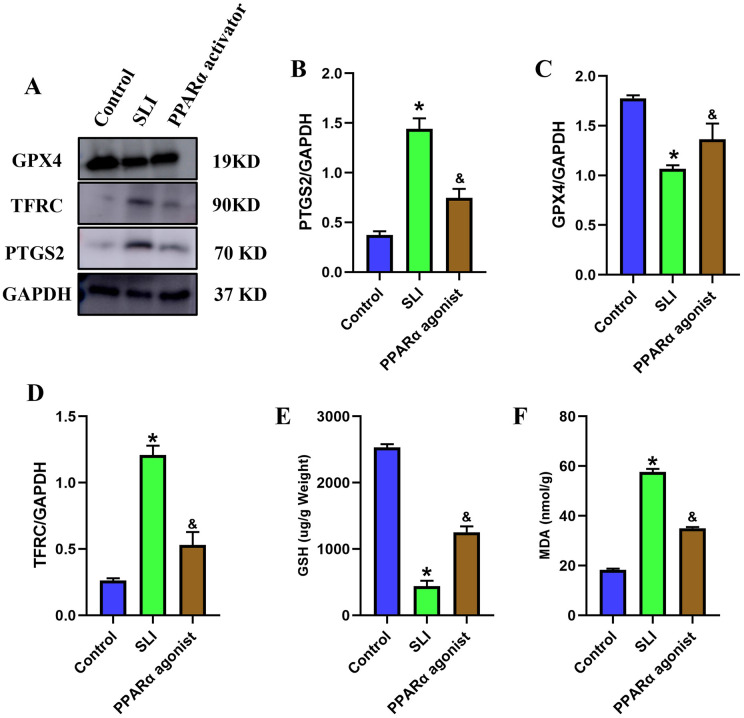
PPARα significantly inhibited the LPS-induced hepatic ferroptosis in SLI mice. (A-D) The protein level of PTGS2, TFRC and GPX4 (n = 3). (E) The level of glutathione (GSH) (n = 3). (F) The level of malondialdehyde (MDA) (n = 3). *, p < 0.05, compared with control group; &, p < 0.05, compared with SLI group. Data are shown as mean ± standard deviation (SD), and n represents the number of mice in each group.

Previous literature has confirmed that PPARα, is a molecule related to the ferroptosis process in acute myeloid leukemia [20], thus we speculate that PPARα might also play a role in SLI. To further investigate the impact of PPARα on survival time and hepatic ferroptosis in SLI mice, we selected the PPARα activator WY-14643 (GLPBIO, Cat. No.: GC14403) to activate the PPAR signaling pathway [21–23] 30 min before LPS treatment. We found that the survival rate of the SLI group was over 50%, which was obviously lower than that in control group (P < 0.05). Interestingly, the survival rates were significantly increased in the PPARα activator group compared to the SLI group (P < 0.05) (S4 Fig). Subsequently, we examined a variety of ferroptosis-related markers and observed an increase in levels of PTGS2, TFRC, and MDA, along with a decrease in level of GPX4 and GSH (Fig 4, P < 0.05) after LPS treatment compared to control group. These results indicate that LPS induces hepatic ferroptosis. Nevertheless, treatment of PPARα activator WY-14643 reversed this trend by reducing intracellular MDA, PTGS2, and TFRC levels, while increasing GSH and GPX4 levels. In summary, our results suggest that downregulation of PPARα mediated the LPS-induced hepatic ferroptosis in SLI.

### Determination of specific cell subtypes mainly expressing PPARα in single-cell data set of liver tissue

**Fig 5:**
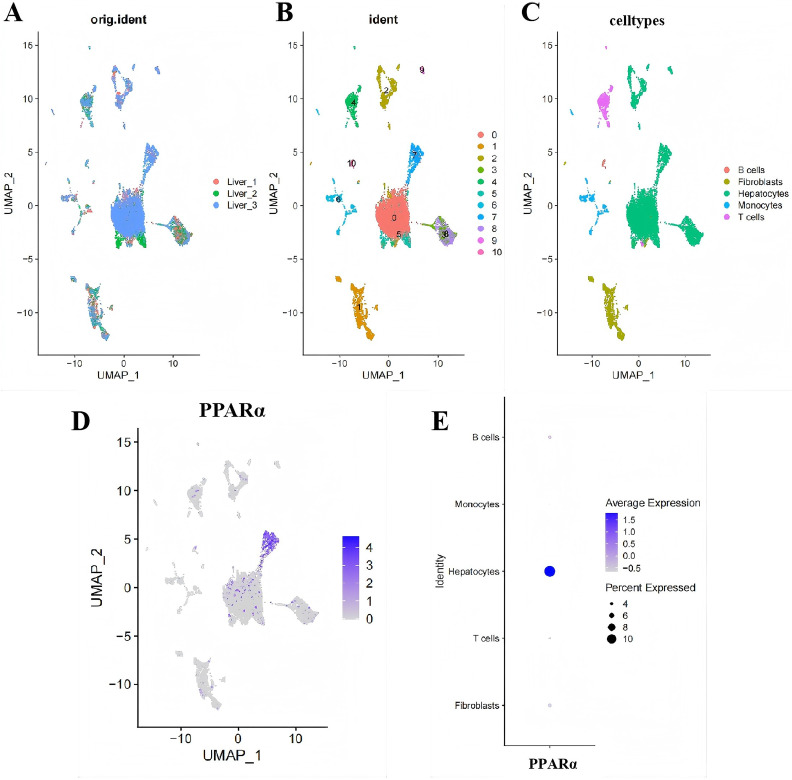
The cell distribution of PPAR α in liver tissues. (A) Uniform Manifold Approximation and Projection (UMAP) plot of each sample. (B) UMAP plot of liver cells allocated into 11 clusters from the liver tissues of 3 mice. (C) UMAP plots of liver cells colored by cell type. (D) Feature plot showing the cell distribution of PPARα in liver tissues. (E) Dotplot plot showing the cell distribution of PPARα in liver tissues.

For the determination of specific cell subtypes predominantly expressing PPARα in single-cell data set of liver tissue, 3 single-cell samples were included in the study (GSE238000). After quality control (QC), 46,794 high-quality cells were included in our analysis (S5 Fig). Subsequently, we used an unbiased UMAP method to group the cells into clusters. We found that the cell distribution among different samples was comparatively uniform, suggesting no significant batch effect existed among samples, which could be utilized for following analysis (Fig 5A). Afterward, all of included cells were clustered into 11 clusters (Fig 5B). Based on the molecular characteristics of each cluster, diverse cell types were successfully annotated using the SingleR package. As displayed in Fig 5C, a total of 5 cell types can be obtained, including epithelial, macrophage and T cells. In order to make sure the annotation accuracy of SingleR method, we computed the expression scores of classical cell markers (epithelial cells: KRT5, KRT14, S100A2, EPCAM; fibroblasts: COL1A1, COL3A1, DCN, LUM; vascular mural: RGS5, MCAM, ACTA2, TAGLN; endothelial cells: PECAM1, AQP1, VWF, COL15A1; T cells: CD3D, CD4, CD8A, TRAC; B cells: MS4A1, CD79A, CD74, CD19; plasma cells: JCHAIN, MZB1, IGHG1, IGHG3; myeloid cells: LYZ, CD68, MS4A6A, CXCL8; mast cells: TPSAB1, KIT, CPA3, TPSB2). The results from manual annotation were consistent with the results from SingleR method. Finally, we examined the cell distribution of PPARα in hepatic tissue and found that it’s predominantly expressed in hepatocytes (Fig 5D, E). This result indicated that downregulation of PPARα probably promotes the progression of SLI through inducing hepatocyte ferroptosis.

### PPARα significantly inhibited the LPS-induced hepatocyte ferroptosis

**Fig 6:**
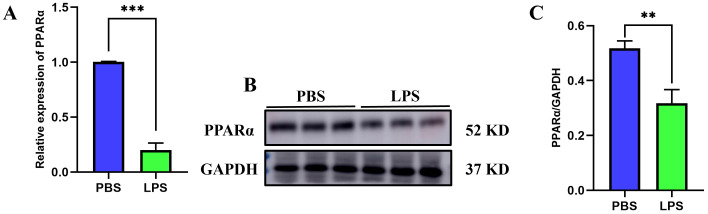
The expression of PPAR α in hepatocytes. (A) The mRNA level of PPARα (n = 3). (B-C) Western blot showing the protein level of PPARα (n = 3). **, p < 0.01, compared with PBS group; ***, p < 0.001, compared with PBS group. Data are shown as mean ± standard deviation (SD), and n represents the number of technical replicates in each group.

**Fig 7:**
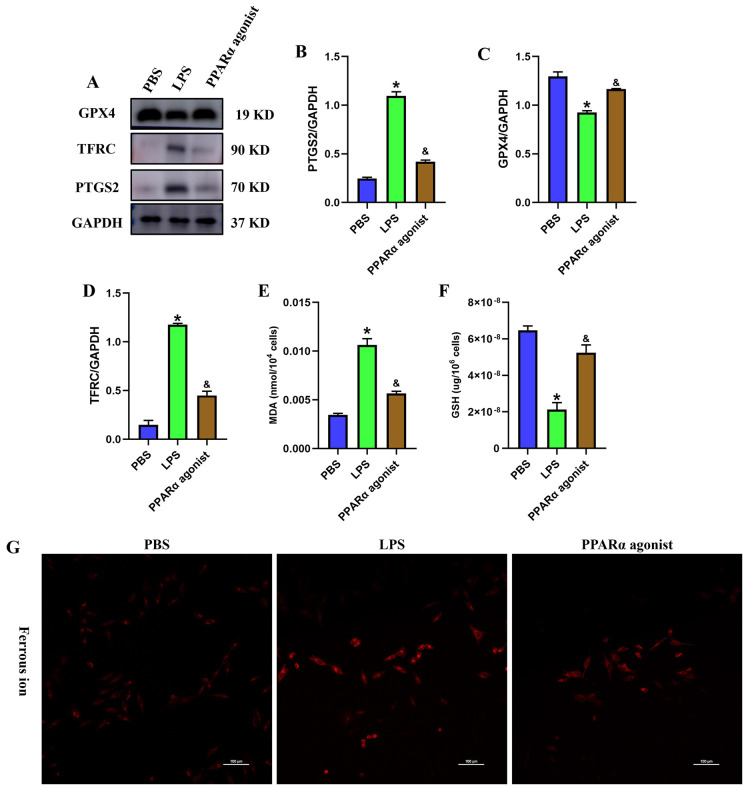
PPARα significantly inhibited the LPS-induced hepatocyte ferroptosis. (A-D) The protein level of PTGS2, TFRC and GPX4 (n = 3). (E) The intracellular level of malondialdehyde (MDA) (n = 3). (F) The intracellular level of glutathione (GSH) (n = 3). (G) The intracellular level of Fe^2+^. Scar bar = 100 um. *, p < 0.05, compared with PBS group; &, p < 0.05, compared with LPS group. Data are shown as mean ± standard deviation (SD), and n represents the number of technical replicates in each group.

**Fig 8:**
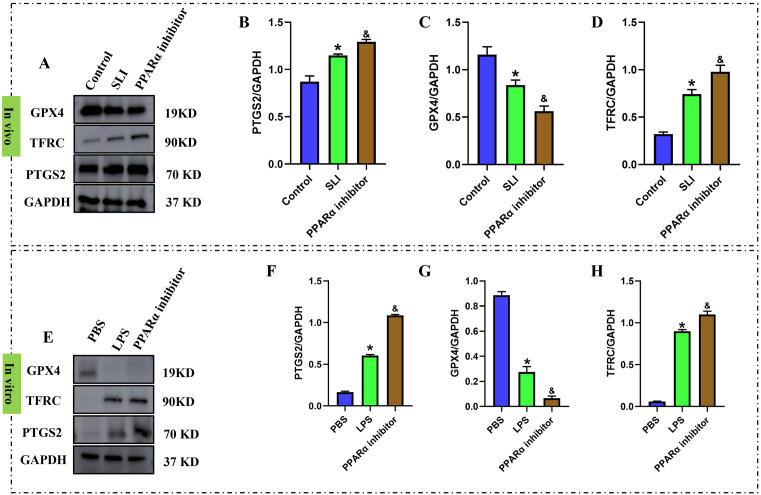
PPARα inhibitor significantly promotes the LPS-induced hepatocyte ferroptosis. (A-D) The protein level of PTGS2, TFRC and GPX4 (n = 3) in hepatic tissue. (E-H) The protein level of PTGS2, TFRC and GPX4 (n = 3) in hepatocyte. *, p < 0.05, compared with Control/PBS group; &, p < 0.05, compared with SLI/LPS group. Data are shown as mean ± standard deviation (SD), and n represents the number of technical replicates in each group.

To validate the above hypotheses, we first measured the expression of PPAR-α in THLE-2 hepatocytes from the control and LPS group using RT-qPCR and western blot. The results of our study confirmed a reduced expression of PPARα in the LPS-treated THLE-2 cells compared to the control group (P < 0.05) (Fig 6). To further reveal the influence of PPARα on ferroptosis in LPS-treated hepatocytes, we treated THLE-2 cells using PPARα activator WY-14643. Next, we evaluated the change in ferroptosis-related marker levels, including MDA, Fe^2+^, GSH content, and the expression levels of PTGS2, TFRC, GPX4 in THLE-2 cells treated with LPS alone or in combination with WY-14643. The results displayed that LPS treatment resulted in significantly upregulated expression levels of MDA, Fe^2+^, PTGS2, TFRC and decreased levels of GSH and GPX4 when compared to the control group (P < 0.05) (Fig 7). However, after the addition of PPARα activator, WY-14643, to the LPS group, the expression levels of MDA, Fe^2+^, TFRC and PTGS2 were downregulated, and the levels of GSH and GPX4 were upregulated in THLE-2 cells (P < 0.05) (Fig 7). Our findings demonstrated that PPARα activator WY-14643 significantly alleviated LPS-induced ferroptosis in THLE-2 cells. In addition to agonist treatment, we performed experiments using a PPARα inhibitor (GW6471), and observed that inhibition of PPARα promotes the occurrence of hepatocyte ferroptosis (P < 0.05) (Fig 8).

## Discussion

This study uncovers a previously unrecognized role of PPARα in promoting SLI. We present strong evidence showing a significant decrease in the expression of PPARα in the livers of LPS-treated mice. PPARα agonist notably alleviated LPS-induced hepatic inflammation and ferroptosis. Additionally, we demonstrated that treatment with the PPARα inhibitor GW6471 in mice and hepatocyte promotes ferroptosis following LPS. Thus, this study identifies PPARα as a key factor in septic liver dysfunction and suggests it as a potential therapeutic target for treating sepsis and liver injury.

Sepsis, an intractable complication caused by severe trauma, shock, or infection, is one of the crucial reasons of mortality in patients in ICUs [24,25]. It’s characterized by multi-organ injury, proinflammatory cytokine release, altered circulation, and oxidative stress [26]. The liver is a primary organ that defends against bacterial infections by eliciting an inflammatory response. Once hepatic dysfunction and damage occurs after sepsis, the impaired liver probably results in severe systemic inflammatory responses, then causing disease progression and even death [27,28]. Although some progress has been obtained in the research field of SLI, its pathogenesis remains primarily unclear. A growing number of studies have revealed that ferroptosis exerts a crucial role in sepsis and SLI [29–31]. Thus, by employing bioinformatic and single cell analysis, this study analyzed the gene expression patterns of liver tissue in SLI mice and healthy mice to screen hub genes that are differently expressed and involved in ferroptosis.

Bioinformatics techniques can process a large quantities of gene expression data, validate hub biomarkers, and offer a theoretical foundation for early treatment and precision intervention. Through integrating multiple data sources, this technique can identify promising biomarkers related to diseases. Thus, bioinformatics techniques are increasingly being utilized in sepsis research. As an effective method of bioinformatic analysis, microarray assay can screen novel molecular markers of disease and understand genetic changes in disease development, which has been validated to be applicable in the study of sepsis-related biomarkers and organ injury [32]. In this work, three databases GSE23767, GSE40180, and GSE104342 were included by screening the gene expression data of SLI in the GEO database. By employing limma R package, we performed differential analysis and obtained 104 DEGs between liver tissues in SLI mice and control ones, including 75 downregulated and 29 upregulated genes. Subsequently, 51 FRDEGs were screened out following functional and pathway enrichment analysis. GO functional analysis displayed that these genes were greatly associated with negative regulation of apoptotic process, the endoplasmic reticulum, and identical protein binding. KEGG pathway analysis exhibited that they were mainly involved in the PPAR signaling pathway. Based on these findings, we concluded that PPAR signaling pathway might represent critical pathway in SLI.

PPARs, a group of nuclear receptor protein molecules, are able to act as transcription factors impacting the expression of downstream genes. PPAR family members comprised three isoforms: PPARα, PPARβ, and PPARγ. The expression level of these three isoforms is different in different organs and tissues, with PPARα being most abundant in the liver. PPARα plays a vital role in regulating oxidative stress by the regulation of gene expression directly through binding to specific downstream response elements [33,34]. Under normal contexts, PPARα can regulate the expression of target genes related to lipid metabolism [35] in tissues with high oxidative rates, such as liver. PPARα impacts lipid metabolism via multiple routes that promote the transport of fatty acids into mitochondria [36]. Under pathological contexts, the overexpression or activation of PPARα can effectively decrease plasma triglycerides, inhibit hepatic steatosis, and relieve adiposity, thus recovering insulin sensitivity [37,38]. In this study, we also found that PPARα was significantly downregulated in the liver from SLI mice compared to control mice. This downregulation demonstrated that PPARα is a novel and promising therapy target in the treatment of SLI.

Recently, more studies have suggested that PPARα is a potential ferroptosis inhibitor in many diseases. For example, Wu et al demonstrated that PPARα downregulation promoted the progression of IgA nephropathy by inducing ferroptosis in human mesangial cells [39]. Hu et al indicated that PPAR-α activation improves myocardial ischemia/reperfusion damage by blocking ferroptosis via upregulating 14-3-3η [40]. Liu et al validated that PPARα can exert an inhibitory effect on dehydroepiandrosterone (DHEA)-induced ferroptosis in granulosa cells by upregulating the expression level of FADS2 [41]. Xing et al identified the PPARα as a new regulator in alleviation of iron overload‐induced ferroptosis in mouse liver [42]. However, no reports on the function of PPARα in sepsis-induced hepatic ferroptosis are available. In the in vivo study, we found that ferroptosis was significantly activated by LPS based on various ferroptosis-related markers. However, the PPARα agonist WY-14643 has been used to effectively block ferroptosis, and improve disruption of oxidative stress in SLI mice.

Next, to fully elucidate and understand the function of PPARα in sepsis-induced hepatic ferroptosis, we performed single-cell RNA sequencing technology to determine the specific cell subtype mainly expressing PPARα in liver tissue. Recently, the rapid progression of single-cell RNA analysis has offered a novel standpoint for the study of SLI [43]. This method permits us to reveal transcriptomic changes in different cell subtypes at the single cell level, helping us to determine the specific expression of target genes. In the current study, three single-cell samples of healthy liver tissues were included. Afterward, we examined the cell distribution of PPARα in hepatic tissue and found that it’s predominantly expressed in hepatocyte. Subsequently, we examined the expression level of PPARα in THLE-2 cells and demonstrated the its’ downregulation in THLE-2 cells treated by LPS versus PBS. To further elucidate the impact of PPARα on ferroptosis in hepatocytes, we pretreated THLE-2 cells with the PPARα activator WY-14643 prior to LPS treatment. Consistent with the in vivo findings, the PPARα activator WY-14643 significantly mitigated LPS-induced ferroptosis in THLE-2 cells.

Several limitations exist. First, while WY-14643 is a selective PPARα agonist, it also exhibits slight activation of PPARγ. Therefore, genetically modified mice with PPARα upregulation should be utilized to validate the function of PPARα. Secondly, the small sample size likely compromises statistical validity. Therefore, a larger sample should be included in subsequent research. Thirdly, to demonstrate that liver PPARα, rather than PPARα in other tissues (such as the heart), is critical for combating sepsis-related mortality, AAV-mediated specific upregulation of liver PPARα should be employed, followed by observation of changes in survival rates in sepsis. Fourthly, although PPAR signaling pathway was identified with a high score and PPAR alpha is most abundant in the liver, it does not necessarily mean other PPAR do not play a role. Examination on the abundance of 3 PPARs should be examined accompanied by application of specific agonists and/or inhibitors. Fifthly, justification of ferroptosis by only testing the level of MDA, GSH, and PTGS2 is insufficient. Transmission electron microscopy should be used to obtain direct evidence of ferroptosis.

## Conclusions

In conclusion, our findings demonstrated that the activation of PPARα probably preserve hepatocytes against sepsis via blocking ferroptosis. Therefore, the PPARα is probably an effective therapeutic target for SLI.

## Supporting information

S1 Table PLOSOne_Human_Subjects_Research_Checklist. (DOCX)

S2 Table Differentially expressed genes. (XLSX)

S3 Table The intersection of ferroptosis-related genes with DEGs. (XLSX)

S4 Table GO enrichment analysis of 51 FRDEGs. (XLSX)

S5 Table KEGG enrichment analysis of 51 FRDEGs. (XLSX)

S1 Fig Original WB blots. (PDF)

S2 Fig The immunohistochemical results from Human Protein Atlas database showing the expression level of three PPAR isoforms in the hepatic tissue. (PDF)

S3 Fig The expression of PPARβ/δ and PPARγ in SLI mice and hepatocytes. (PDF)

S4 Fig Survival curve. (PDF)

S5 Fig Quality control of scRNA-seq data from 3 hepatic samples. (PDF)
