# Supplementary figures and images for: Multi-omics analysis identifies PPARα as a key inhibitor of hepatocyte ferroptosis in sepsis-associated liver injury
Source: PLoS One. 2026 Feb 19;21(2):e0338591. doi: 10.1371/journal.pone.0338591 (PMC12919794; doi:10.1371/journal.pone.0338591)

**Fig 3 (E)**

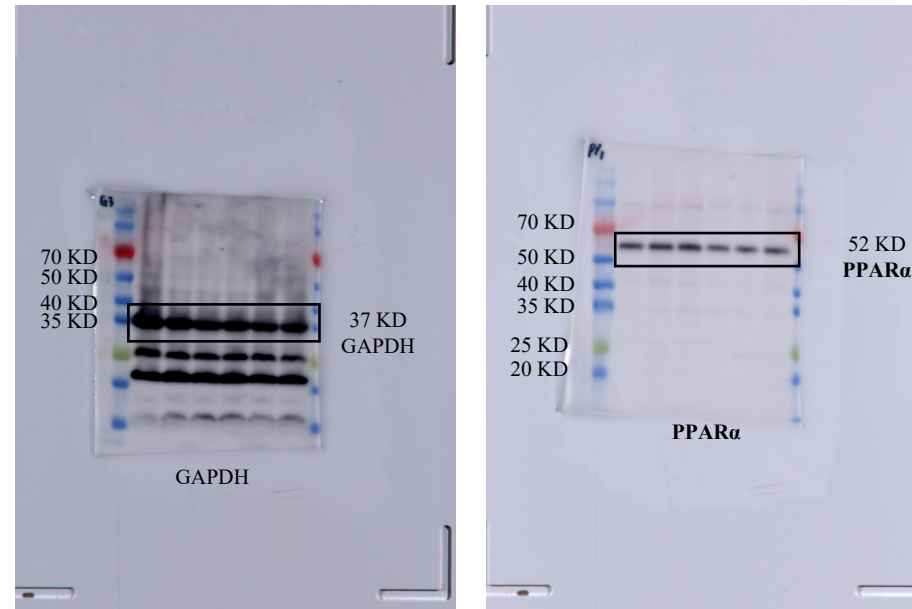

**Fig 4 (B)**

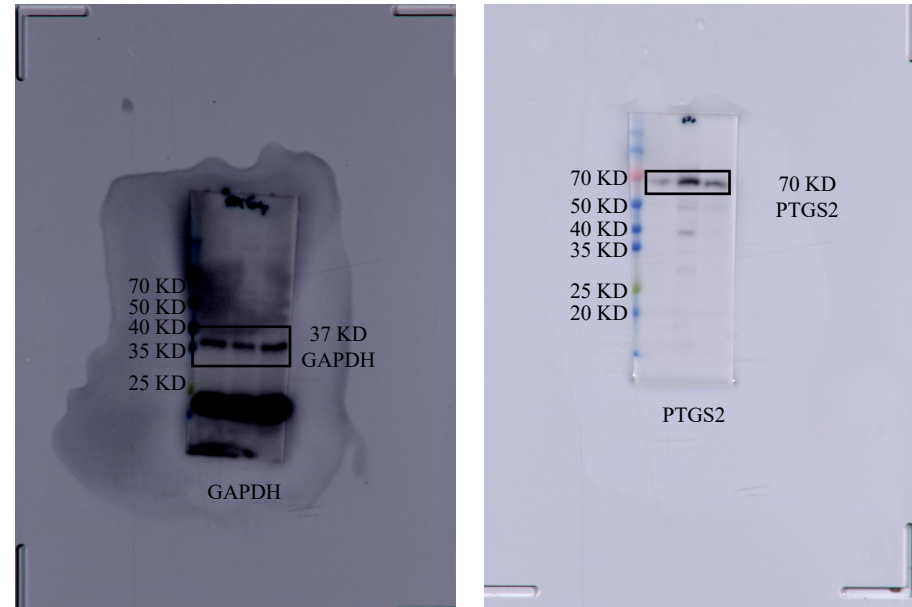

**Fig 6 (B)**

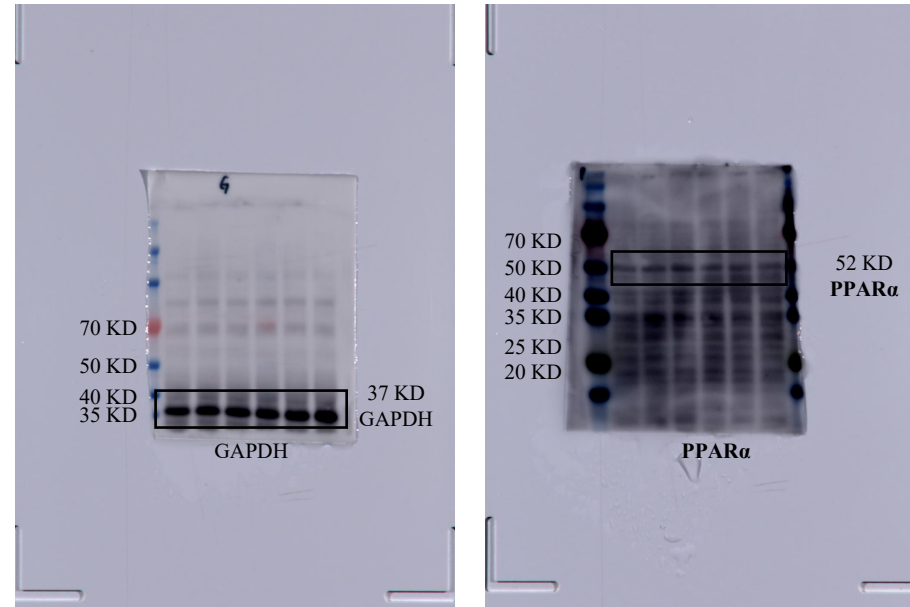

**Fig 7 (B)**

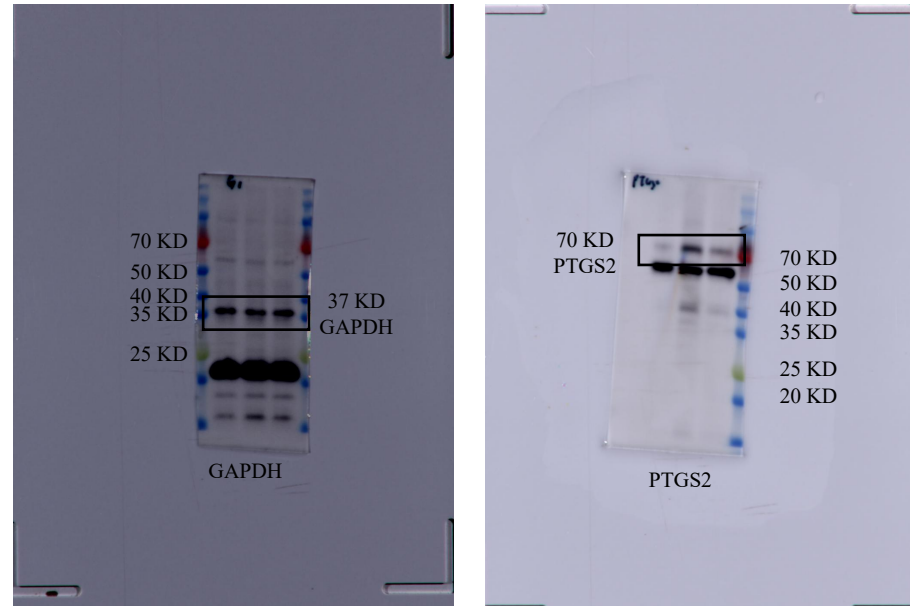

Supplement: S1 Fig — (PDF) [file pone.0338591.s006.pdf]

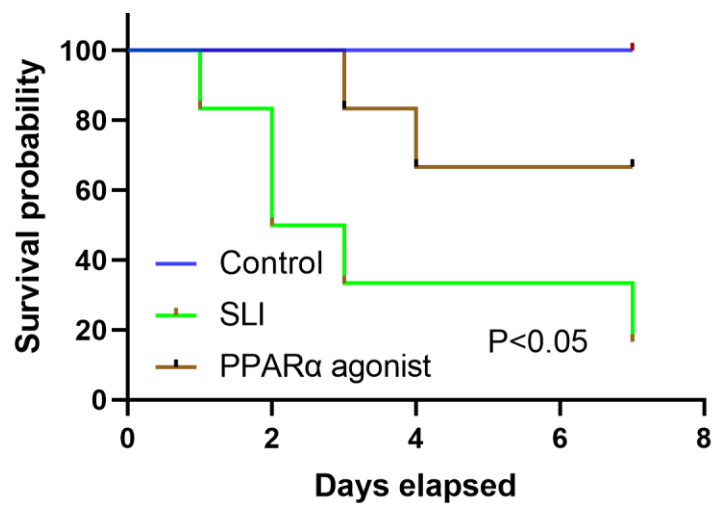

Supplementary Fig 4 Survival curve.

Supplement: S4 Fig — (PDF) [file pone.0338591.s009.pdf]

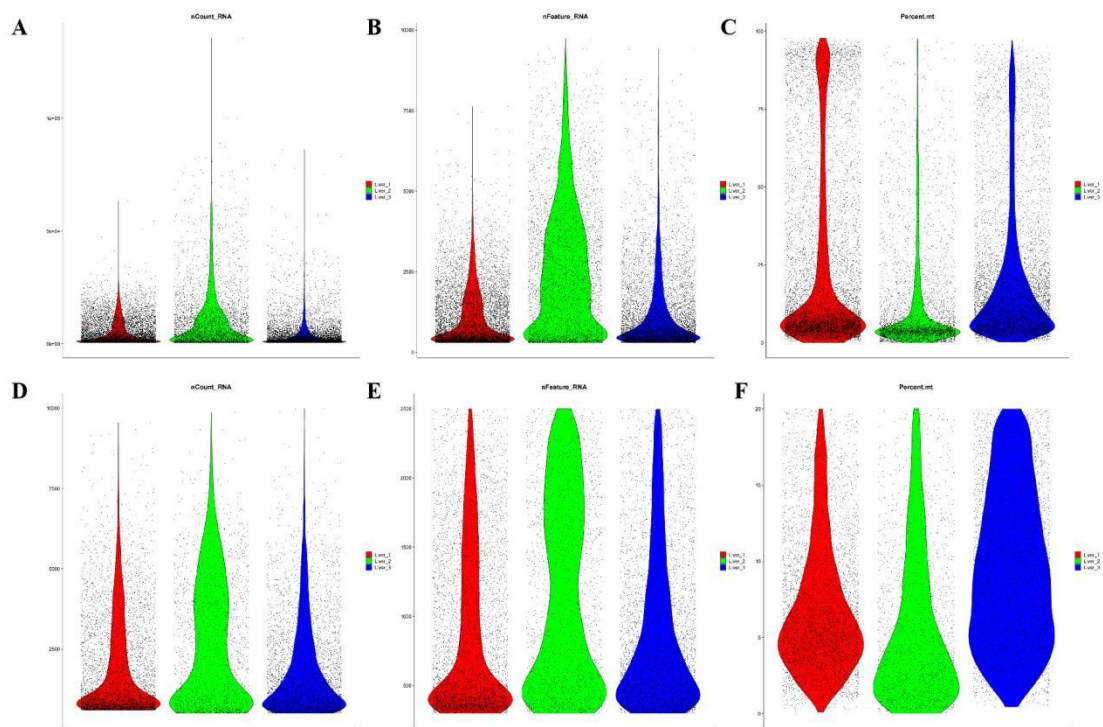

Supplementary Fig 5 Quality control of scRNA-seq data from 3 hepatic samples.

Supplement: S5 Fig — (PDF) [file pone.0338591.s010.pdf]
