# Supplementary material for: Multi-omics analysis identifies PPARα as a key inhibitor of hepatocyte ferroptosis in sepsis-associated liver injury
Source: PLoS One. 2026 Feb 19;21(2):e0338591. doi: 10.1371/journal.pone.0338591 (PMC12919794; doi:10.1371/journal.pone.0338591)
Supplement: S2 Fig — (PDF) [file pone.0338591.s007.pdf]

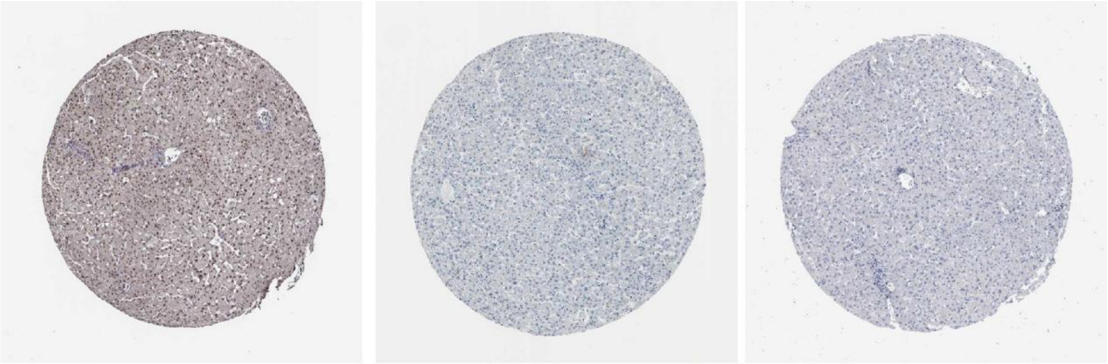

PPARα PPARγ PPARβ

Supplementary Fig 2 The immunohistochemical results from Human Protein Atlas database showing the expression level of three PPAR isoforms in the hepatic tissue.
