# Supplementary material for: Multi-omics analysis identifies PPARα as a key inhibitor of hepatocyte ferroptosis in sepsis-associated liver injury
Source: PLoS One. 2026 Feb 19;21(2):e0338591. doi: 10.1371/journal.pone.0338591 (PMC12919794; doi:10.1371/journal.pone.0338591)
Supplement: S3 Fig — (PDF) [file pone.0338591.s008.pdf]

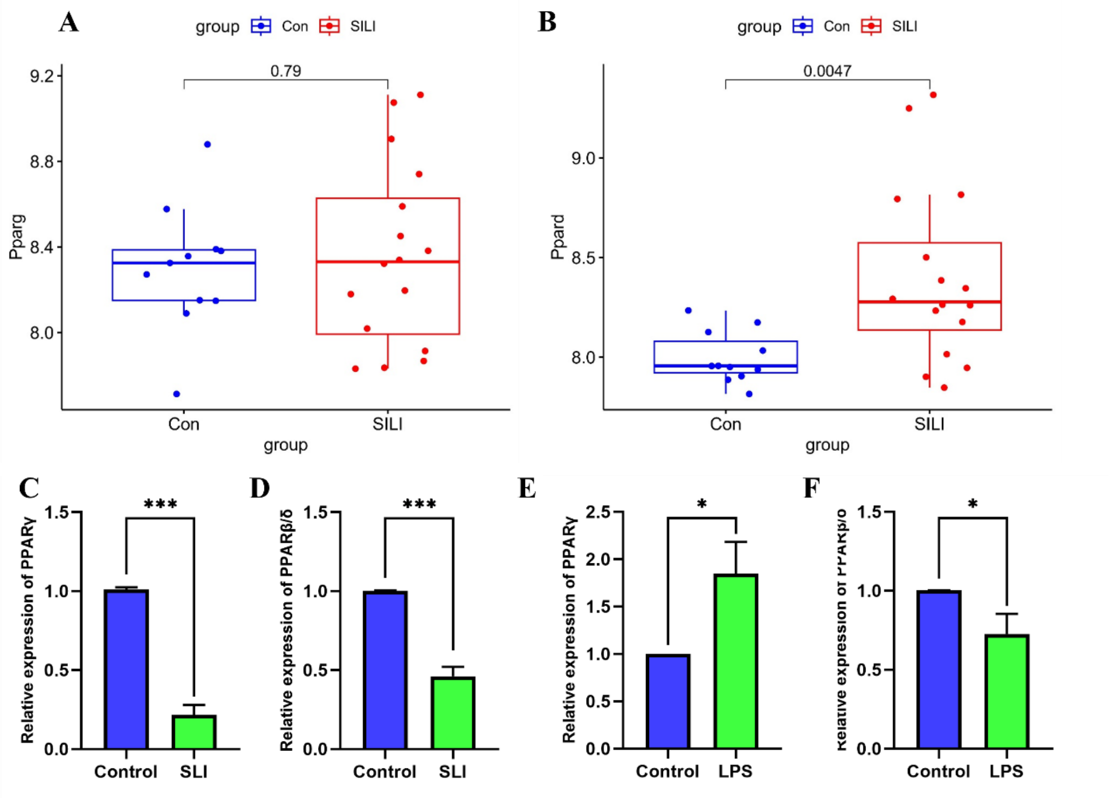

Supplementary Fig 3 The expression of PPARβ/δ and PPARγ in SLI mice and hepatocytes. (A) Expression validation of PPARβ/δ in the integrated dataset. (B) Expression validation of PPARγ in the integrated dataset. (C) The mRNA levels of PPARγ in hepatic tissue. (D) The mRNA levels of PPARβ/δ in hepatic tissue. (E) The mRNA levels of PPARγ in hepatocytes. (F) The mRNA levels of PPARβ/δ in hepatocytes. *, $p<0.05$, compared with Control group. ***, $p<0.001$, compared with Control group. Data are shown as mean ± standard deviation (SD).
